# Value of critical literature analysis for knowledge development

**DOI:** 10.5935/0004-2749.2024-1008

**Published:** 2024-04-03

**Authors:** Newton Kara-Junior

**Affiliations:** 1 Ophthalmology Department, Universidade de São Paulo, São Paulo, SP, Brazil; 2 Editor-in-Chief of Arquivos Brasileiros de Oftalmologia

Not too long ago, medical knowledge was transmitted by word of mouth and supported by clinical experience. Reading books, though important for basic training, is a limited source of knowledge on new developments in diagnostic and therapeutic techniques because the time interval between writing and publishing the book is large compared to the pace at which medicine advances. The most reliable method of updating oneself about medical advancements is through the search, selection, and interpretation of scientific articles available online^(1)^.

Thus, the greatest challenge for doctors to be updated is no longer to have access to information, but rather to acquire the ability to select reliable one. Methodological biases are identified only after scrutinizing the methodology in scientific articles, a skill honed by researchers and lacking in most of the medical community. Thus, doctors must be trained to critically analyze scientific literature to ensure their practice is grounded in the latest scientific evidence, which universities and specializations rarely teach^(2,3)^.

The *sensu stricto* postgraduate course, which traditionally is aimed at those who wish to pursue an academic career in teaching and research, currently enrolls many students who wish to learn how to interpret scientific literature, and thus, stay abreast with the latest medical advances^(3)^.

Knowledge transmitted through expository lectures also gives way to active teaching/learning methodologies, where students search for information outside the classroom. Young “generation Z” doctors who were born in the digital era tend to minimize the importance of teachers and prefer to study alone, accessing knowledge electronically^(4)^.The issue is that in the scientific world there is also fake news. Thus, finding information is easy, but distinguishing reliable from unreliable information is difficult.

Considering these new challenges for knowledge development, I take on the position of Editor-in-Chief of the *Arquivos Brasileiros de Oftalmologia* (ABO), a scientific journal committed to the dissemination of new knowledge.

ABO belongs to the *Conselho Brasileiro de Oftalmologia* (CBO) and is the most important scientific publication in Brazilian ophthalmology. As it was distributed free of charge to CBO members and is open access, ABO is a proponent of open science^(5)^.

Researchers and scholars demand the free dissemination of knowledge, which is respected by national scientific journals^(6)^. In our case, CBO, by assuming all editorial costs, guarantees open access to readers and does not charge authors for publication.

Furthermore, with the diligent work of the editors who have preceded us, such as Profs. Waldemar Belfort, Rubens Belfort, Rubens Belfort Jr, Harley Bicas, Wallace Chamon, and Eduardo Rocha, ABO has become a respectable global journal^(7,8)^; thus, continuing this valuable work will be a formidable task.

As Editor-in-Chief working together with a dedicated team of Associate Editors and an experienced Editorial Board formed by former editors, our action plan for ABO includes further improving the quality of published studies, reducing the interval between receipt and publication of research, and promoting the journal abroad to attract other renowned authors. Moreover, it should teach young authors to conduct research with appropriate methodology and enable readers to critically evaluate scientific articles.

In addition to reviewing the submitted articles, we will provide guidance on how to improve the methodology used, invest in promising topics, and organize immersion courses in scientific research and critical literature analysis, as high-quality and safe medical practice relies on continuous update of medical information.

The most reliable way to acquire medical knowledge is through reviewing the literature in electronic databases. However, there are the following two prerequisites: (1) establish a habit of seeking answers to doubts in scientific journals, and (2) learn to critically evaluate scientific articles^(9)^. Although journals with a high impact factor (IF) submit received studies to competent peer reviewers^(10)^, there is no guarantee that all articles published are of adequate quality. Similarly, good studies may be published in lower IF journals. Thus, readers must know how to critically evaluate scientific articles to avoid basing clinical practice on information that may not represent scientific truth.

The desire to participate in critical literature analysis training should come from the medical community itself. This training enables the reader to recognize the most reliable study designs and identify methodological flaws that may influence study results and conclusions (biases).

One of the most important aspects of publication is the description of the methodology used to obtain the data. In this item, attention must be paid to issues such as: a) the way the sample was prepared (identifying possible selection bias); b) exposure to factors other than the intervention of interest (identify possible conduct bias); and c) losses or exclusions of individuals included in the study (identify possible follow-up bias) or the diagnosis of the outcome (identify possible detection bias), because the abstract or the conclusion of the article should not be valued without verifying if the methodology is adequate.
